# Two Cases of Myelitis

**Published:** 1900-04

**Authors:** Edward B. Angell

**Affiliations:** Rochester, N. Y.; Neurologist to Rochester City Hospital; member of the American Neurological Association


### TWO CASES OF MYELITIS.

ONE, SUBACUTE—RECOVERY; THE OTHER, CENTRAL—DEATH.

By EDWARD B. ANGELL, M. D., Rochester, N. Y.
Neurologist to Rochester City Hospital; member of the American Neurological Association.

INFLAMMATION of the spinal cord, or myelitis, is not an uncommon disease. The clinical picture, however, called up in the mind of most practitioners by this term, is one in which a segment of the cord, more or less extended, is involved throughout its whole thickness. This transverse inflammation of the cord is, indeed, but one of the forms in which it may manifest itself. It must be remembered that the spinal cord is subdivided into more or less definite areas or tracts, some of the functions of which are well defined. For this reason a limited inflamed area will develop a symptomatology very different from that resulting from an inflammation of a transverse segment, or from one involving the structures about the central canal. In many subacute cases the grouping of symptoms approximates so closely those due to the slowly developing structural changes of degeneration, that error in diagnosis may easily follow. The prognosis becomes needlessly grave, and a hopeless therapy lets slip the chance of recovery possible in the milder forms of the inflammatory disease.

It is with a view of calling attention to the necessity of recognising early the different phases of a malady, usually regarded as fatal, that I have ventured to report these two cases—one, a limited subacute myelitis, the other an instance of the overwhelming central form, running its fatal course rapidly and changing its symptoms as it involved more and more of the spinal marrow.

Carl H., a farmer, single, aged about 35 years, entered the Rochester City Hospital, December 6, 1898, suffering with almost complete paraplegia. His history was negative and he denied specific disease, though he admitted the possibility of its acquisition. Five weeks before he had felt a "stitch" in his back. He continued work and it passed away. In a few days he noticed a decided lameness in the lumbar region which soon extended to his legs. The legs began to feel numb and cold and became weak. There was some pain in the right hip and knee, but at no time was pain a marked symptom. Until three weeks prior to his entering the hospital he continued to hobble around, but since then found it impossible to walk. At the time of entrance he had lost control over his bladder and he suffered from retention of urine, requiring catheterisation. Unless he was watchful he had involuntary stools. His legs had become somewhat emaciated. It was difficult for him to stand on his feet; with eyes shut he would sway hopelessly. Even in bed he gave evidence of marked muscle incoördination. The deep reflexes were intensified, both knee jerks being exaggerated, while an ankle clonus could be readily elicited, especially on the right side. Of the superficial reflexes, the plantar and epigastric were retained, the cremaster and abdominal were absent. There was no priapism, nor indeed any erections. The sexual function was abolished. There was some atrophy of the thigh muscles, but only quantitative alteration to electrical stimulation. Sensation to touch and pain was diminished in the legs, especially on the right side. Thermal sensibility was not tested. Over the abdomen, above the umbilicus, sensation was normal; indeed, he asserted that above his legs he was just as well as ever. The temperature was 99°; pulse, 120; respiration, 20. The urine was normal in character. There were no eye indications. Here, then, we had a clinical picture very similar to the one presented by a type of degeneration known as ataxic paraplegia. Paraplegia, incoördination of the leg muscles, some sensory loss below the level of the lesion; absence of eccentric or radiating pains; no marked girdle sensation; increased knee jerks and marked ankle clonus; interference with voluntary control over the vesical and anal sphincters. Associated with these was some loss of muscle in the affected limbs.

Except for its rather rapid development the disease simulated ataxic paraplegia, and doubtless the structures most affected were the areas which become degenerated in the chronic malady. Regarding it as a subacute inflammatory process, possibly due to specific trouble, notwithstanding his protestation of innocence from the malady that lurks in evil places, I placed him upon antipyrin and the iodide of potasium. Antipyrin certainly seems to have some control over congestive conditions of the nervous system. It was given in five grain doses every three hours, while the iodide of potash, well diluted, was increased two grains each dose, until he was taking two drams, three times a day. Later, as he began to improve, electrical exercise of the leg muscles was instituted with beneficial results.

Within a week there were indications of improvement which shortly became rapid and early in January, a month after his entrance, he left the hospital, recovery being well advanced. He could walk with ease, though with some spasticity of gait. He had regained control of the visceral functions, the numbness and coldness of the legs had passed away and he regarded himself well enough to take French leave, unwilling to accept my earnest advice to remain longer in order to insure lasting recovery.

June 12th, he presented himself at my office and gave a good account of the intervening time. He had worked steadily, had regained his normal weight, and aside from subjective numbness, more noticeable in the right leg, regarded himself well. Examination, however, elicited evidence of some limited degenerative changes in the cord which may yet cause trouble. There was a distinct band-like girdle sensation about the right side of the abdomen on a level with the navel; slight atrophy of the right thigh with excessive knee-jerk and right ankle clonus. The cremaster reflex, formerly absent, responded promptly; there was no sensory disturbance nor difficulty in gait. A trace of the former loss of control over the bladder persisted in a spasmodic or jerky urination. Unless the degenerative changes extend, he is practically well and will suffer little inconvenience from the results of the focal myelitis.

A true paraplegic ataxia is very slow in development, requiring months to become pronounced, but when once established the disease can only be regarded as hopeless. Subacute myelitis, on the other hand, is relatively rapid in its onset and in its early stages amenable to treatment. If, however, the acute stage results in permanent anatomical changes, recovery is not complete and progressive degenerative changes, either above or below, may follow in accordance with the pathological law. The other case that I record is of a unique character. The initial trouble was a slowly progressive degeneration or sclerosis of the lateral columns—spasmodic paraplegia. Suddenly there was a marked change in the symptoms and an acute inflammation of the cord developed. It extended rapidly along the central canal, both above and below the site of the original focus of sclerosis and resulted fatally with the development of abnormally high temperature.

November 22, 1899, I saw, in consultation with Dr. McCaulay, Mr. G. N., a married man of 47 years. His family and personal health history were without interest, with the exception of a bad attack of acute inflammatory rheumatism, eleven years previous. From this he never fully recovered, although it did not interfere with his vocation, that of collector. He was beyond the suspicion of having had syphilis, the possibility of which he denied. Early in October, 1896, a year before, he first noticed prickly sensations in the toes and fingers of all extremities. They gradually became more pronounced; his leg muscles became weak, walking an effort and spasmodic in character, with a gradual developing paraplegia. In a year he was no longer able to walk, found it almost impossible to stand and his leg muscles became stiff with easily developed spasm. Incoördination was so pronounced that he "lost his feet in bed" His arms were unaffected. He suffered no pain and mentality was excellent. Appetite and digestion were good, the bowels for the month preceding my visit, obstinately constipated. The urine was normal and control over the sphincters retained. He had lost seventeen pounds in weight. Examination brought out the following symptomatology. He was scarcely able to stand, requiring support, due to the loss of leg control. All the reflexes, both superficial and deep, were exaggerated below the gluteal. There was a marked ankle clonus in both legs and both knee jerks were very excessive. Above the gluteal the reflexes were unaffected. The leg muscles were stiff and rigid, presenting the phenomena known as "clasp knife" rigidity. They were slightly atrophied. Sensation was unaffected and motor power in the legs was fairly good, he being able to resist passive movements of some energy. The muscular sense was absolutely *nil*; with his eyes closed he could not tell where his feet were when placed one above the other; he really lost their position in bed. Eye examination gave negative results; indeed, above the umbilicus all reactions were normal.

A diagnosis of lateral sclerosis of the mid-dorsal region was made and no hope of improvement could be given. Within a few days he experienced a sudden onset of acute girdle pain about the umbilical region—a sense of severe, unbearable constriction. This was somewhat relieved by poultices and persisted only for two nights. He rapidly became weaker, retention of urine developed and his mind became dull.

December 16th, he was admitted to the Rochester City Hospital, and reëxamination showed marked change in the objective symptoms. There was greater weakness in the thigh muscles, which were flabby and wasted in place of the previous rigidity. All reflexes below the gluteal were exaggerated as before. The gluteal and epigastric reflexes had vanished. Insensibility to all impressions was dulled up to the umbilicus. The hand grip was still strong on both sides. There was a decided blunting of the intelligence. He had retention of urine and obstinate constipation. In the course of a week the temperature became elevated, with marked noisy delirium and the paralysis below the umbilicus was increased. All the reflexes of the trunk and lower extremities were abolished and anesthesia developed, its area extending upward as the upper segments of the cord became involved, until its upper border reached mid-sternum. The bowels moved involuntarily and unconsciously. There was spasmodic involuntary flow of urine, except when drawn by the catheter. At no time, however, did the urine become alkaline. On the eleventh day of admission, coma took the place of delirium, the pupils both became equally contracted, the paralysis, both motor and sensory, became complete, and the temperature rapidly rose, reaching at the time of death on the thirteenth day after admission 108°.

At the autopsy, twelve hours after death, the cord was examined from the first cervical to the mid-dorsal region. The dura was not involved, but there was acute capillary injection of the pia throughout its course and the arterioles penetrating the substance of the cord were markedly distended. Throughout the length of the cord there was great venous congestion. The cord itself was soft and had lost its normal form. Cross section at different levels showed decided softening, especially of the lateral columns in the mid-dorsal region, less so in the cervical enlargement. Unquestionably the mid-dorsal region was the seat of the initial inflammatory action, which extended both upward and downward, involving the whole structure of the cord, but most marked throughout the lateral tracts. In the brain, the dura showed evidence of old adhesions, but no recent pathological change. The pia, however, was in a state of acute inflammation with organising lymph over both frontal lobes, although the congestion of the pial vessels was most marked in the region of the medulla, pons and ventricles. Otherwise there was no intracerebral lesion, nor any evidence of cerebritis.

In this case we have an instance of an acute inflammatory process, developing in a tract which was the seat of a well-established sclerosis. For what cause I cannot say. I can only venture the opinion that at the time of the development of acute eccentric pains about the umbilicus, a small hemorrhage had occurred in the degenerated area. The onset of the pain was sudden and therefore probably due to a vascular cause. The irritation thus set up induced an acute myelitis in the sclerosed areas, which shortly became central and extended rapidly up and down the whole length of the cord, even involving the cerebral membranes. As it extended downward and affected the lumbar centers, muscular rigidity gave place to flaccid paralysis, and the reflexes once exaggerated became absolutely lost. The involvement of the cervical region will explain the abnormally high temperature and contracted pupils, while the injected meningeal vessels of the cerebrum undoubtedly caused the active delirium.

These two cases illustrate the extremes of inflammatory action in the cord. Myelitis, on the one hand, may be light, limited in the area involved and successfully treated. On the other hand, it may be insidious in its beginning, then develop almost explosively and result in a rapidly fatal termination, involving the whole central nervous system.

295 ALEXANDER STREET.

---

MME. JENNY LIND-GOLDSCHMIDT, the great cantatrice, has left by will $30,000 to the Samaritan Hospital at Stockholm.

